# The chemokine, CXCL12, is an independent predictor of poor survival in ovarian cancer

**DOI:** 10.1038/bjc.2012.49

**Published:** 2012-03-13

**Authors:** A Popple, L G Durrant, I Spendlove, P Rolland, I V Scott, S Deen, J M Ramage

**Affiliations:** 1 Academic Department of Clinical Oncology, Molecular Medical Sciences, City Hospital Campus, University of Nottingham, Nottingham NG5 1PB, UK; 2 Department of Obstetrics and Gynaecology, Royal Derby Hospital, Derby DE22 3NF, UK; 3 Division of Histopathology, University Hospitals Nottingham, Nottingham NG7 2UH, UK

**Keywords:** chemokines, CXCL12, CXCR4, ovarian cancer

## Abstract

**Background:**

The chemokine CXCL12 and its cognate receptor, CXCR4, have been implicated in numerous tumour types where expression promotes tumour growth, angiogenesis, metastasis and suppresses tumour immunity.

**Methods:**

Using a tissue microarray of 289 primary ovarian cancers coupled to a comprehensive database of clinicopathological variables, the expression of CXCL12 and CXCR4 was assessed by immunohistochemistry and its impact in terms of survival and clinicopathological variables was determined.

**Results:**

Patients whose tumours expressed high levels of CXCL12 had significantly poorer survival (*P*=0.026) than patients whose tumours failed to produce this chemokine. Lack of CXCL12 expression within tumours was associated with a 51-month survival advantage for patients when compared with patients whose tumours expressed high levels of CXCL12. FIGO stage, adjuvant chemotherapy and the absence of macroscopic disease after surgery were all shown to predict prognosis independently of each other in this cohort of patients. CXCL12 was independently predictive of prognosis on multivariate analysis (*P*=0.016). There was no correlation between CXCL12 and any clinicopathological variable.

**Conclusion:**

The chemokine CXCL12 is an independent predictor of poor survival in ovarian cancer. High expression of CXCL12 was seen in only 20% of the tumours, suggesting a role for anti-CXCL12/CXCR4 therapy in the management of these patients.

Ovarian cancer is the fifth leading cause of cancer among women and the leading cause of mortality from gynaecological cancers (Parkin *et al*, 1999), with epithelial cancer being responsible for 90% of ovarian malignancies. It can be classified into four major categories: serous, mucinous, endometrioid and clear cell. Each subtype has different clinical, molecular and biological characteristics and may represent different diseases (Gomez-Raposo *et al*, 2010). However, the main cause of treatment failure and death is metastasis. The chemokines involved in lymphocyte homing and migration can also be used by tumour cells to metastasise. In a study of expression of 14 chemokine receptors, only CXCR4 was expressed within ovarian cancer cell lines (Scotton *et al*, 2001). The only CXCR4 ligand, CXCL12, induced migration, integrin expression, proliferation and invasion. CXCL12 was abundantly expressed within biopsy (Scotton *et al*, 2002) and ascite samples from ovarian cancer patients.

CXCL12 (stromal derived factor-1), a 68-amino-acid chemokine (8 kDa) belonging to the CXC chemokine family, is constitutively expressed in the bone marrow and in other tissues, including the skin, heart, liver, lung and brain endothelium (Dar *et al*, 2006). This constitutive expression is responsible for trafficking and localisation of immature and maturing leucocytes to these tissues, suggesting a role in immune surveillance (Bleul *et al*, 1996). It is also a potent costimulator of helper T cells (Nanki and Lipsky, 2000). CXCL12 can also act as an anti-inflammatory chemokine by promoting the polarisation of helper T cells to become antigen-specific regulatory cells (Meiron *et al*, 2008).

CXCR4 is a 352-amino-acid rhodopsin-like seven transmembrane G protein-coupled receptor (Furusato *et al*, 2010) used for HIV entry (Feng *et al*, 1996). The CXCL12/CXCR4 axis has an important role in the regulation of stem/progenitor cell trafficking (Peled *et al*, 1999). AMD3100 inhibits CXCL12/CXCR4 interactions and has been approved as a drug for hematopoietic stem cell mobilisation from the bone marrow to the blood for autologous stem cell transplantation (DiPersio *et al*, 2009a, 2009b). Endothelial cells can express both CXCL12 and CXCR4 and are key regulators of angiogenesis (Salcedo and Oppenheim, 2003).

Various tumours, in particular the androgen-dependent tumours such as prostate, breast and ovarian cancers, produce CXCL12 and express CXCR4 (Zhou *et al*, 2001; Taichman *et al*, 2002; Sun *et al*, 2003; Kukreja *et al*, 2005). In these tumours, CXCL12 can have pleiotropic roles in autocrine growth stimulation as a tumour cell chemoattractant (Muller *et al*, 2001; Begley *et al*, 2005; Akashi *et al*, 2006; Engl *et al*, 2006; Sun *et al*, 2007) and as an endothelial stem cell attractant contributing to tumour vascularisation (Orimo *et al*, 2005), and can suppress tumour immunity (Zhou *et al*, 2001; Meiron *et al*, 2008). Furthermore, expression of CXCR4 and CXCL12 predicts lymph node metastasis in colorectal (Poznansky *et al*, 2000), oesophageal (Gockel *et al*, 2006) and breast cancer (Liu *et al*, 2010). CXCR4 has been shown to be a predictor of poor survival in nasopharyngeal carcinoma (Wang *et al*, 2005), renal cell carcinoma (Wang *et al*, 2009), gastrointestinal tumours (Schimanski *et al*, 2008) and ovarian cancer (Jiang *et al*, 2006).

Although the CRCR4/CXCL12 axis has been implicated in ovarian cancer biology, there have been limited studies on its prognostic value. Using tissue microarray technology (TMA) we analysed a large cohort of ovarian tumour samples from 289 patients for CXCL12 and CXCR4 expression. The results demonstrate that expression of high levels of CXCL12 is an independent marker of poor prognosis in ovarian cancer.

## Patients and Methods

We followed the reporting recommendations for tumour marker prognostic studies (REMARK) (McShane *et al*, 2005) when compiling this manuscript. This work was approved by the Derby Royal Hospital Ethics Committee.

### Patient study and clinicopathological variables

A total of 360 patients with ovarian cancer were entered into this study, and consisted of patients undergoing a laparotomy for primary ovarian cancer. Information on cancer size, stage, presence or absence of residual disease after surgery, histological type and grade, age at diagnosis, and type of adjuvant treatment was collected for all patients (Table 1). Histological material was available for analysis in 339 cases. The paraffin-embedded tissue blocks from these patients were dated from 1 January 1984 until 31 December 1997. Disease-specific survival was calculated from the operation date until 31 November 2005, when any remaining survivors were censored. The database was audited to ensure validity; there were no major discrepancies with >97% of data available.

### Patient characteristics

During the study period, patients with high-grade stage I and stage II–IV disease received chemotherapy. The specific chemotherapy varied but reflected the best current practice; most recently, this treatment was platinum based. In detail, 95 patients received no adjuvant therapy, 77 had non-platinum chemotherapy, 77 platinum, 87 carboplatin, and 8 carboplatin and taxol chemotherapy; 1 patient received taxol alone and 5 patients received radiotherapy. In all, 62 patients participated in the International Collaborative Group for Ovarian Neoplasia trials I–IV, during which the allocated chemotherapy was randomised. Although the study spans a 14-year period, there was no significant change in the survival of patients treated in the earlier or latter part of the study. This is in line with the unaltered survival of ovarian cancer patients over the last 30 years (Bjorge *et al*, 1998).

### Tissue microarrays and immunohistochemistry

Tissue microarrays were constructed as described previously. Immunohistochemical analysis was performed using a routine streptavidin–biotin peroxidase method. Tissue-array sections were first deparaffinised with xylene, rehydrated through graded alcohol and immersed in methanol containing 0.3% hydrogen peroxide for 20 min. In order to retrieve antigenicity, sections were immersed in pH 9.0 EDTA buffer and heated in an 800-W microwave for 10 min at high power and 10 min at low power. Endogenous avidin/biotin binding was blocked (avidin/biotin blocking kit, Vector Labs, Peterborough, UK), followed by addition of 100 *μ*l 1 : 5 normal swine serum (NSS) to Tris-buffered saline (TBS) for 15 min to block non-specific binding.

Sections were incubated with either 100 *μ*l of mouse polyclonal antibody (MAB350; 1 : 50 dilution) recognising human CXCL12 (R&D Systems) or a mouse monoclonal antibody (MAB172; 1 : 600 dilution; R&D Systems, Abingdon, UK) recognising human CXCR4. Positive controls were whole sections of colorectal cancer tissue. The primary antibody was omitted from the negative control.

After washing with TBS, sections were incubated with 100 *μ*l of biotinylated goat anti-mouse/rabbit immunoglobulin (Dako Ltd, Ely, UK) diluted 1 : 100 in NSS, for 30 min, washed in TBS and incubated with 100 *μ*l of pre-formed streptavidin–biotin/horseradish peroxidase complex (Dako) for 60 min. Visualisation was achieved using 3, 3′-diaminobenzidine tetra hydrochloride (DAB, Dako), and then lightly counterstained with haematoxylin (Dako), dehydrated in alcohol, cleared in xylene (Genta Medica, York, UK), and mounted with distyrene, plasticiser and xylene (DPX–BDH, Poole, UK).

### Evaluation of staining

The tumour cores were first imaged using a NanoZoomer (Hamamatsu, Bridgewater, NJ, USA). The cores were assessed at × 20 magnification by two observers, both with experience in the analysis of TMAs. Tumours were classified by H scores and assessed for high, moderate, low and negative CXCL12 and CXCR4 expression. Both observers were blinded to clinical and pathological parameters. In the few cases (<5%) where there was a discrepancy, a review was performed and a consensus reached.

### Statistical analysis

Statistical analysis was performed using the SPSS package (version 16 for Windows, SPSS Inc., Chicago, IL, USA). Pearson's *χ*^2^-tests were used to determine the significance of associations between categorical variables. Disease-specific survival calculations included all patients whose death related to ovarian cancer, whereas deaths from other causes were censored at the time of death. Kaplan–Meier curves were used to assess factors that influenced survival. The statistical significance of differences in disease-specific survival between groups was estimated using the log-rank test. The Cox proportional-hazards model was used for multivariate analysis to determine the relative risk and independent significance of individual factors. *P*-values <0.05 were considered statistically significant.

## Results

### Clinical and pathological data

In the cohort of ovarian cancer patients, the mean age at diagnosis was 61 years (range 24–90). Using the current Surveillance, Epidemiology and End Results Program age categorisation system, at diagnosis 59% of the patients were >60 years, 40% were 30–60 years and only 2 of 357 were <30 years. Serous carcinoma was the commonest histological type (49%) followed by undifferentiated (15%), endometrioid (12%), mucinous cystadenocarcinoma (10%), clear cell (7%) and other types (7%). All patients were treated surgically, of which 42% had their masses optimally debulked (residual tumour less than 2 cm) with no macroscopic disease left. In all, 49% were in stage III, 26% in stage I, 11% in stage IV and 11% in stage II. Histological grading of the serous carcinomas showed that 90% were high grade (160) and 10% low grade (18). The other tumours were graded as 27% well differentiated, 11% moderately differentiated and only 6% poorly differentiated. Patients’ characteristics are summarised in Table 1.

### CXCL12 expression in ovarian cancer tissue

**Figure 1:**
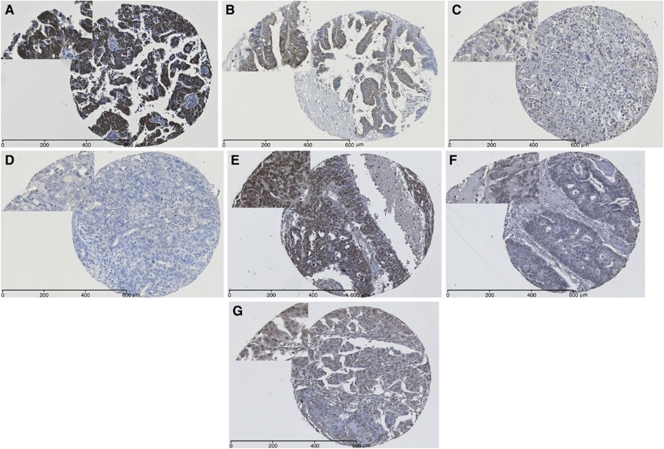
Photomicrographs of ovarian TMA cores immunohistochemically stained for CXCL12 (**A–D**) and CXCR4 (**E–G**). The level of CXCL12 expression ranged from (**A**) high, (**B**) moderate, (**C**) weak to (**D**) negative. The level of CXCR4 expression ranged from (**E**) high, (**F**) moderate to (**G**) low.

Analysis of CXCL12 was possible in 289 of the 360 cores (80%), with the remainder not available on the cut slide, being lost during antigen retrieval or not demonstrating viable tumour cells in the core. Owing to the loss of cores, the clinicopathological data for the 289 samples were assessed to confirm that they represented the original cohort (Table 1). CXCL12 staining was seen in the cytoplasm of tumour cells (Figures 1A–D). Of the 289 samples, 199 (69%) tumours were positive for CXCL12. The X-tile program (Yale University, CT, USA) was used to determine cut-points to divide the samples into low/negative, moderate and high groups using a H-score of 0–3 (low; 39%), 3–90 (moderate; 41%) and 90–300 (high; 20%) for expression of CXCL12 in tumours. The X-tile program was also used to divide the cohort into two groups: low/high groups of H-score 0–10 (low; 55%) and 10–300 (high; 45%) for CXCL12 tumour expression.

### CXCR4 expression in ovarian cancer tissue

Analysis of CXCR4 was possible in 241 of the 360 cores (67%), with the remainder not available on the cut slide, being lost during antigen retrieval or not demonstrating viable tumour cells in the core. Owing to the loss of cores, the clinicopathological data for the 241 samples were assessed to confirm that they represented the original cohort (Table 1). CXCR4 staining was seen in the nucleus of tumour cells (Figures 1E–G). Of the 241 samples, none were negative for CXCR4 in tumour cells. The majority of tumours had moderate CXCR4 expression and accounted for 49% of the cohort. The X-tile program provided cut-points to divide tumours into low, moderate and high groups with H-score 0–60 (low; 11%), 60–115 (moderate; 20%) and 115–300 (high; 69%) for tumour CXCR4 expression.

### Increased CXCL12 expression but not CXCR4 expression reduces patients survival

**Figure 2:**
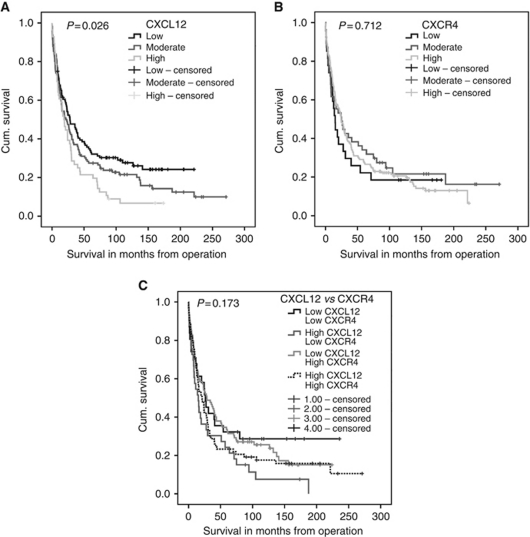
Kaplan–Meier plots for disease-specific survival for (**A**) CXCL12 (low (0–3) moderate (3–90) and high (90–300)) and (**B**) CXCR4 (low (0–60) moderate (60–115) and high (115–300)) and (**C**) a combination of CXCL12 and CXCR4 expression. CXCL12 as low/high groups, using X-tile, of H-score 0–10 (low) and 10–300 (high) *vs* CXCR4 as low/high groups, using X-tile, of H-score 0–110 (low) and 110–300 (high).

Kaplan–Meier plots were used to analyse the relationship between expression levels of CXCL12 and CXCR4 and disease-specific survival. In all, 235 patient samples were analysed for both markers. Increasing expression of CXCL12 within tumours significantly reduces patient survival (*P*=0.026) (Figure 2). Patients with low CXCL12 expression lived a mean of 75.9 months, compared with 59.1 months for moderate expression and 24.2 months for high expression (Table 2). When comparing differing levels of CXCR4 expression (Figure 2B) with disease-specific survival, no difference was found (*P*=0.712). Patients with low CXCR4 expression lived a mean of 47.5 months, compared with 74.6 months for moderate expression and 58.1 months for high expression (Table 2). When tumour cell expression of high and low CXCL12 was compared with high and low CXCR4 expression (Figure 2C), in patients who showed both low CXCL12 and CXCR4 expression 32 of 235 patients (32%) had the best survival of 82 months, and patients with high CXCL12 and low CXCR4 displayed the worst survival, with 14 of 235 patients (33%) having a survival time of only 39 months. However, overall there was no linear correlation between CXCL12 and CXCR4 (*P*=0.409 (data not shown)) and expression patterns showed no significant effects on survival of ovarian cancer patients (*P*=0.173).

### No correlation with clinicopathological features and CXCL12 and CXCR4 expression

The relationship between CXCL12 and CXCR4 expression within ovarian tumours and standard clinicopathological variables was measured using the Pearson's *χ*^2^-test. Expression of both CXCL12 and CXCR4 was not significantly correlated with any of the clinicopathological variables, including stage, grade, type, residual disease or type of chemotherapy (Table 3).

### Expression of CXCL12 by ovarian tumours is an independent prognostic marker

In order to determine the relative influence of CXCL12 and CXCR4 on other patient and tumour variables, known to affect prognosis, a multivariate analysis was performed using the Cox multivariate regression model. The variables included were those that have been shown to be significantly related to disease-specific survival on univariate analysis (macroscopic residual, adjuvant therapy and FIGO (International Federation of Gynecology and Obstetrics) stage). In this model, macroscopic residual disease (*P*<0.001), adjuvant therapy (*P*=0.001) and FIGO stage (*P*<0.001) were seen to retain independent prognostic significance (Table 4). Expression of CXCL12 by ovarian tumours was shown to be an independent prognostic marker (*P*=0.016). Expression of CXCR4 by ovarian tumours was not shown to be independent of macroscopic residual disease, adjuvant therapy and FIGO stage (*P*=0.364; Table 5).

## Conclusion

CXCL12 has multiple roles in tumour pathogenesis by promoting tumour growth, enhancing tumour angiogenesis, suppressing tumour immunity and participating in tumour metastasis via expression of CXCR4 (Kryczek *et al*, 2007). In this study, TMA was used to assess the expression of CXCL12 and CXCR4 in relation to clinico-pathological characteristics and overall survival of 289 ovarian cancer patients. This study has the advantage of over 14 years of patient follow-up. CXCL12 and CXCR4 expression was seen in 69% and 100% of patient samples, respectively. This study demonstrated reduced disease-specific survival (*P*=0.026) in ovarian cancer patients whose tumours showed moderate or high CXCL12 expression. There was a 51-month survival advantage for patients whose tumours lacked CXCL12 expression, when compared with patients whose tumours expressed high levels of this chemokine. Furthermore, CXCL12 expression was an independent predictor of poor survival and was equally expressed by all ovarian tumour types. These data contrast with a smaller immunohistochemistry study by Jiang *et al* (2006) using 80 patients with a shorter follow-up (median 37 months). CXCL12 was detected in 40/44 (91% of patients); however, in this study CXCL12 did not correlate with disease survival. This contrasted with our study, which analysed 289 patients with a median follow-up of 167 months (range 95–335 months). We used X-tile to define our cutoff and divided CXCL12 expression into three groups (low (0–3), moderate (3–90) and high (90–300)). Using these criteria we were able to show a graded decrease in survival with increased CXCL12 expression. In the Jiang study groups were divided using two cutoffs (using IRS scoring instead of a H-score) and showed no significance. We also analysed our data in two groups: low (0–10) and high (10–300), and showed significance (data not shown, *P*=0.019).

Published data would suggest that 80% of the ovarian carcinomas are of serous type (Soslow, 2008). As undifferentiated carcinomas are believed to be of serous type, the total number of serous carcinomas in this study was 69%. The difference in this distribution may be due to the availability of tissues as some biopsies from serous carcinomas in advanced stage were not suitable for TMA. As the results for CXCL12 were independent of histological type, the lower number of serous carcinomas in this study should not affect the results.

In contrast to CXCL12, the expression of CXCR4 within our study had no significant effect on patient survival (*P*=0.712) or correlation with any other clinicopathological variable. This is in contrast with other studies that showed that CXCR4 expression was an independent prognostic factor for poor survival in epithelial ovarian cancer patients (Jiang *et al*, 2006). These studies used lower numbers of patients with shorter follow-up than our study. One explanation for the differences in the effect of CXCR4 on survival between the studies may be the localisation of CXCR4 within the cell. CXCR4 expression is not confined to the cell surface and the CXCR4 receptor may be internal until activation/signalling occurs, whereby the receptor becomes present at the cell surface, allowing interaction with CXCL12 and activating signalling cascades (Forster *et al*, 1998). In our study, 100% of the tumours showed nuclear staining, with 69% strong nuclear and cytoplasmic staining. However, in the Jiang study, CXCR4 expression was only seen within the cytoplasm of 59% of patients. This may be a reflection of the differing antigen retrieval methods showing greater or less sensitivity of detection.

As the CXCR4/CXCL12 pathway is involved in the metastasis of tumour cells, this has important implications for the survival of ovarian cancer patients where the main cause of death is attributed to metastasis. Indeed, in breast cancer high levels of expression of CXCL12 were shown in organs representing the first destination for breast cancer metastasis (Muller *et al*, 2001). However, we have demonstrated high levels of CXCL12 expression at the primary site of the tumour to be involved in poor prognosis. This raises the question whether chemokines may not just work at target organs but could also be involved in the departure of cells from the primary tumours. Indeed, high levels of CXCL12 have been shown to be fugetactic to lymphocytes (Poznansky *et al*, 2000) and a B16 melanoma engineered to produce CXCL12 at high levels was found to be chemo-repellent to antigen-specific T cells (Vianello *et al*, 2006). Alternatively, CXCL12 stimulates the production of metalloproteases, which are important in local tumour invasion (Majka *et al*, 2000; Samara *et al*, 2004).

CXCL12 regulation within breast and ovarian tumours has been attributed to oestradiol, which activates oestrogen receptors and induces the production of CXCL12 by tumour cells (DiPersio *et al*, 2009a, 2009b). Over 70% of ovarian and breast cancers overexpress the oestrogen receptor. The binding of CXCL12 to its receptor CXCR4 is thought to induce proliferation of tumour cells. In this way, CXCL12 mediates the cancer cell proliferation action of oestrogen and may account for the poor prognosis of patients with high CXCL12 (Hall and Korach, 2003).

Hypoxia has also been shown to induce CXCL12 expression by primary human ovarian tumour cells where hypoxia-inducible factor-1 is the central mediator (Kryczek *et al*, 2005). Kryczek *et al* studied CXCL12 in ovarian tumours in association with VEGF and found that hypoxia synchronously induces tumour CXCL12 and VEGF production (Kryczek *et al*, 2005). Hypoxia-induced signals would be an important factor for initiating and maintaining an active synergistic angiogenic pathway mediated by CXCL12 and VEGF. Our group has previously demonstrated that high VEGF expression occurs in a small proportion of ovarian cancers (7%) and this independently predicts poor prognosis (Duncan *et al*, 2008). However, there was no correlation between CXCL12 and VEGF expression in this series (data not shown).

CXCL12 has contradictory roles in immune responses. During homeostasis, tissues constitutively express CXCL12, which promotes leucocyte extravasation and immune surveillance. However, normal ovaries fail to secrete CXCL12 and it is only induced by pro-inflammatory mediators such as IL-1 and TNF*α* and during hypoxia (Kukreja *et al*, 2005). Under conditions of inflammation CXCL12 can downregulate immune responses (Karin, 2010). It can polarise helper T cells, macrophages and plasmacytoid dendritic cells to secrete the immunoregulatory cytokine IL-10 (Zhou *et al*, 2001; Meiron *et al*, 2008). CXCL12 can also promote TNF*α* expression, which can activate nuclear factor *κ*B (NF-*κ*B) and act back on tumour cells to induce cell surface CXRC4 expression. In effect, TNF*α* can amplify the CXCL12 signal (Kulbe *et al*, 2005), and therefore neutralising TNF*α*, neutralising CXCL12 (Karin, 2010), antagonising CXCR4 (Lapteva *et al*, 2005; Liang *et al*, 2007; Du *et al*, 2008; Kajiyama *et al*, 2008) or inhibiting NF-κB (Miyanishi *et al*, 2010) may be an effective therapy for ovarian cancer.

In conclusion, this study has shown that expression of CXCL12 is an independent prognostic indicator of poor survival in ovarian cancer. Furthermore, it identifies a group of patients (20%) with high CXCL12 expression in which targeting the CXCL12/CXCR4 axis may be an effective target.

## Figures and Tables

**Table 1:** Clinicopathological variables for the patient cohort (*n*=360) and cores stained for both CXCL12 (289) and CXCR4 (*n*=241)

**Variable**	**Categories**	**Frequency of total cohort (%)**	**Frequency of the CXCL12-stained cohort (%), *n*=289**	**Frequency of the CXCR4-stained cohort (%), *n*=241**
SEER age characteristics (*n*=357)	<30 years at diagnosis 30–60 years at diagnosis >60 years at diagnosis	2 (1) 143 (40) 212 (59)	2 (1) 112 (39) 172 (60)	1 (0.4) 92 (38) 146 (61)
				
Macroscopic residual disease (*n*=348)	Absent	143 (40)	116 (40)	95 (39)
	Present	201 (56)	161 (56)	136 (56)
				
FIGO stage	I	95 (26)	77 (27)	62 (26)
	II	38 (11)	32 (11)	27 (11)
	III	175 (49)	141 (49)	118 (49)
	IV	40 (11)	30 (10)	26 (11)
	Unknown	12 (3)	9 (3)	8 (3)
				
Histological type	Serous carcinoma	178 (49)	148 (51)	128 (53)
	Mucinous cystoadenocarcinoma	35 (10)	29 (10)	22 (9)
	Endometrioid	42 (12)	35 (12)	30 (12)
	Clear cell	25 (7)	20 (7)	18 (7)
	Undifferentiated	54 (15)	39 (13)	31 (13)
	Others	24 (7)	18 (6)	11 (5)
				
Serous tumour grade	High	160 (44)	131 (45)	113 (47)
	Low	18 (5)	17 (6)	15 (6%)
				
Tumour grade of all other tumours	Well differentiated (3)	98 (27)	75 (26)	64 (27)
	Moderately differentiated (2)	39 (11)	33 (11)	26 (11)
	Poorly differentiated (1)	20 (6)	16 (6)	12 (5)
	Unknown	21 (6)	14 (5)	9 (4)
				
Adjuvant therapy (*n*=356)	No	101 (28)	78 (27)	67 (28)
	Yes	249 (69)	204 (71)	168 (70)

Abbreviation: FIGO=International Federation of Gynecology and Obstetrics.

**Table 2:** Mean survival time in relation to either CXCL12 and CXCR4 expression

	**Mean survival time (months) in relation to CXCL12 expression**				**Mean survival time (months) in relation to CXCR4 expression**			
		**95% confidence interval**				**95% confidence interval**		
**Expression**	**Estimate (months)**	**Lower bound**	**Upper bound**	***P*-value**	**Estimate (months)**	**Lower bound**	**Upper bound**	***P*-value**
Low	75.9	59.1	92.6	0.026	47.5	22.8	72.1	0.712
Moderate	59.1	45.5	72.6		74.6	45.6	103.7	
High	24.2	13.9	34.5		58.1	46.5	69.7	
Overall	66.6	55.3	77.8		65.1	53.1	77.1	

**Table 3:** Univariate analysis of CXCL12 and CXCR4 expression in correlation with standard clinicopathological variables using the *χ*^2^-test

	***χ*^2^-test (*P*-value)**	
**Variable**	**CXCL12**	**CXCR4**
Tumour FIGO stage	0.184	0.603
Tumour grade	0.865	0.239
Macroscopic residual disease	0.769	0.141
Adjuvant therapy	0.144	0.704
Histological type	0.347	0.658

Abbreviation: FIGO=International Federation of Gynecology and Obstetrics.

*P*-values <0.05 are accepted to be significant.

**Table 4:** Multivariate analysis of CXCL12 compared with FIGO stage, residual disease and adjuvant therapy

		**95% CI for Exp(*B*)**		
	**Exp(*B*)**	**Lower**	**Upper**	***P*-value**
*FIGO stage*				
Stage 1	1			<0.001
Stage 2	2.483	1.370	4.501	
Stage 3	5.639	3.235	9.829	
Stage 4	6.047	3.168	11.543	
				
*Macroscopic residual disease*				
Absent	1			<0.001
Present	2.002	1.374	2.917	
				
*Adjuvant therapy*				
No	1			<0.001
Yes	0.493	0.331	0.735	
				
*CXCL12*				
Low	1			0.016
Moderate	1.215	0.892	1.655	
High	1.684	1.180	2.404	

Abbreviations: CI=confidence interval; FIGO=International Federation of Gynecology and Obstetrics.

The analysis is based on Cox multivariate regression model.

*P*-values <0.05 are accepted to be significant.

**Table 5:** Multivariate analysis of CXCR4 compared with FIGO stage, residual disease and adjuvant therapy

		**95% CI for Exp(*B*)**		
	**Exp(*B*)**	**Lower**	**Upper**	***P*-value**
*FIGO stage*				
Stage 1	1			<0.001
Stage 2	2.633	1.391	4.986	
Stage 3	5.638	3.120	10.189	
Stage 4	5.698	2.878	11.281	
				
*Macroscopic residual disease*				
Absent	1			<0.002
Present	1.928	1.284	2.894	
				
*Adjuvant therapy*				
No	1			<0.001
Yes	0.471	0.309	0.719	
				
*CXCR4*				
Low	1			0.364
Moderate	0.667	0.381	1.166	
High	0.796	0.505	1.257	

Abbreviations: CI=confidence interval; FIGO=International Federation of Gynecology and Obstetrics.

The analysis is based on Cox multivariate regression model.

*P*-values <0.05 are accepted to be significant.

## References

[bib1] Akashi T, Koizumi K, Nagakawa O, Fuse H, Saiki I (2006) Androgen receptor negatively influences the expression of chemokine receptors (CXCR4, CCR1) and ligand-mediated migration in prostrate cancer DU-145. Oncol Rep 16: 831–836 16969502

[bib2] Begley L, Monteleon C, Shah R, MacDonald J, Macoska J (2005) CXCL12 overexpression and secretion by aging fibroblasts enhance human prostrate epithelial proliferation *in vitro*. Aging Cell 4: 291–298 16300481 10.1111/j.1474-9726.2005.00173.x

[bib3] Bjorge T, Engeland A, Hansen S, Trope CG (1998) Prognosis of patients with ovarian cancer and borderline tumours diagnosed in Norway between 1954 and 1993. Int J Cancer 75: 663–670 9495231 10.1002/(sici)1097-0215(19980302)75:5<663::aid-ijc1>3.0.co;2-x

[bib4] Bleul CC, Fuhlbrigge RC, Casasnovas JM, Aiuti A, Springer TA (1996) A highly efficacious lymphocyte chemoattractant, stromal cell-derived factor 1 (SDF-1). J Exp Med 184: 1101–1109 9064327 10.1084/jem.184.3.1101 PMC2192798

[bib5] Dar A, Kollet O, Lapidot T (2006) Mutual, reciprocal SDF-1/CXCR4 interactions between hematopoietic and bone marrow stromal cells regulate human stem cell migration and development in NOD/SCID chimeric mice. Exp Hematol 34: 967–975 16863903 10.1016/j.exphem.2006.04.002

[bib6] DiPersio JF, Micallef IN, Stiff PJ, Bolwell BJ, Maziarz RT, Jacobsen E, Nademanee A, McCarty J, Bridger G, Calandra G (2009a) Phase III prospective randomized double-blind placebo-controlled trial of plerixafor plus granulocyte colony-stimulating factor compared with placebo plus granulocyte colony-stimulating factor for autologous stem-cell mobilization and transplantation for patients with non-Hodgkin's lymphoma. J Clin Oncol 27: 4767–4773 19720922 10.1200/JCO.2008.20.7209

[bib7] DiPersio JF, Stadtmauer EA, Nademanee A, Micallef IN, Stiff PJ, Kaufman JL, Maziarz RT, Hosing C, Fruehauf S, Horwitz M, Cooper D, Bridger G, Calandra G (2009b) Plerixafor and G-CSF versus placebo and G-CSF to mobilize hematopoietic stem cells for autologous stem cell transplantation in patients with multiple myeloma. Blood 113: 5720–5726 19363221 10.1182/blood-2008-08-174946

[bib8] Du YF, Shi Y, Xing YF, Zeng FQ (2008) Establishment of CXCR4-small interfering RNA retrovirus vector driven by human prostate-specific antigen promoter and its biological effects on prostate cancer *in vitro* and *in vivo*. J Cancer Res Clin Oncol 134: 1255–1264 18431597 10.1007/s00432-008-0394-2 PMC12161737

[bib9] Duncan TJ, Al-Attar A, Rolland P, Scott IV, Deen S, Liu DT, Spendlove I, Durrant LG (2008) Vascular endothelial growth factor expression in ovarian cancer: a model for targeted use of novel therapies? Clin Cancer Res 14: 3030–3035 18483368 10.1158/1078-0432.CCR-07-1888

[bib10] Engl T, Relja B, Marian D, Muller I, Ringel E, Beecken W, Jones D, Blahata R (2006) CXCR4 chemokine receptor mediates prostrate tumour cell adhesion through alpha5 and beta3 integrins. Neoplasia 8: 290–301 16756721 10.1593/neo.05694 PMC1600676

[bib11] Feng Y, Broder CC, Kennedy PE, Berger EA (1996) HIV-1 entry cofactor: functional cDNA cloning of a seven-transmembrane, G protein-coupled receptor. Science 272: 872–877 8629022 10.1126/science.272.5263.872

[bib12] Forster R, Kremmer E, Schubel A, Breitfeld D, Kleinschmidt A, Nerl C, Bernhardt G, Lipp M (1998) Intracellular and surface expression of the HIV-1 coreceptor CXCR4/fusin on various leukocyte subsets: rapid internalization and recycling upon activation. J Immunol 160: 1522–1531 9570576

[bib13] Furusato B, Mohamed A, Uhlen M, Rhim JS (2010) CXCR4 and cancer. Pathol Int 60: 497–505 20594270 10.1111/j.1440-1827.2010.02548.x

[bib14] Gockel I, Schimanski CC, Heinrich C, Wehler T, Frerichs K, Drescher D, von Langsdorff C, Domeyer M, Biesterfeld S, Galle PR, Junginger T, Moehler M (2006) Expression of chemokine receptor CXCR4 in esophageal squamous cell and adenocarcinoma. BMC Cancer 6: 290 17176471 10.1186/1471-2407-6-290 PMC1766934

[bib15] Gomez-Raposo C, Mendiola M, Barriuso J, Hardisson D, Redondo A (2010) Molecular characterization of ovarian cancer by gene-expression profiling. Gynecol Oncol 118: 88–92 20439111 10.1016/j.ygyno.2010.03.012

[bib16] Hall JM, Korach KS (2003) Stromal cell-derived factor 1, a novel target of estrogen receptor action, mediates the mitogenic effects of estradiol in ovarian and breast cancer cells. Mol Endocrinol 17: 792–803 12586845 10.1210/me.2002-0438

[bib17] Jiang YP, Wu XH, Shi B, Wu WX, Yin GR (2006) Expression of chemokine CXCL12 and its receptor CXCR4 in human epithelial ovarian cancer: an independent prognostic factor for tumor progression. Gynecol Oncol 103: 226–233 16631235 10.1016/j.ygyno.2006.02.036

[bib18] Kajiyama H, Shibata K, Terauchi M, Ino K, Nawa A, Kikkawa F (2008) Involvement of SDF-1alpha/CXCR4 axis in the enhanced peritoneal metastasis of epithelial ovarian carcinoma. Int J Cancer 122: 91–99 17893878 10.1002/ijc.23083

[bib19] Karin N (2010) The multiple faces of CXCL12 (SDF-1alpha) in the regulation of immunity during health and disease. J Leukoc Biol 88: 463–473 20501749 10.1189/jlb.0909602

[bib20] Kryczek I, Lange A, Mottram P, Alvarez X, Cheng P, Hogan M, Moons L, Wei S, Zou L, Machelon V, Emilie D, Terrassa M, Lackner A, Curiel TJ, Carmeliet P, Zou W (2005) CXCL12 and vascular endothelial growth factor synergistically induce neoangiogenesis in human ovarian cancers. Cancer Res 65: 465–472 15695388

[bib21] Kryczek I, Wei S, Keller E, Liu R, Zou W (2007) Stromal-derived factor (SDF-1/CXCL12) and human tumour pathogenesis. Am J Physiol 292: C987–C995 10.1152/ajpcell.00406.2006 16943240

[bib22] Kukreja P, Abdel-Mageed AB, Mondal D, Liu K, Agrawal KC (2005) Up-regulation of CXCR4 expression in PC-3 cells by stromal-derived factor-1alpha (CXCL12) increases endothelial adhesion and transendothelial migration: role of MEK/ERK signaling pathway-dependent NF-kappaB activation. Cancer Res 65: 9891–9898 16267013 10.1158/0008-5472.CAN-05-1293

[bib23] Kulbe H, Hagemann T, Szlosarek PW, Balkwill FR, Wilson JL (2005) The inflammatory cytokine tumor necrosis factor-alpha regulates chemokine receptor expression on ovarian cancer cells. Cancer Res 65: 10355–10362 16288025 10.1158/0008-5472.CAN-05-0957

[bib24] Lapteva N, Yang AG, Sanders DE, Strube RW, Chen SY (2005) CXCR4 knockdown by small interfering RNA abrogates breast tumor growth *in vivo*. Cancer Gene Ther 12: 84–89 15472715 10.1038/sj.cgt.7700770

[bib25] Liang Z, Brooks J, Willard M, Liang K, Yoon Y, Kang S, Shim H (2007) CXCR4/CXCL12 axis promotes VEGF-mediated tumor angiogenesis through Akt signaling pathway. Biochem Biophys Res Commun 359: 716–722 17559806 10.1016/j.bbrc.2007.05.182 PMC1986788

[bib26] Liu Y, Ji R, Li J, Gu Q, Zhao X, Sun T, Wang J, Li J, Du Q, Sun B (2010) Correlation effect of EGFR and CXCR4 and CCR7 chemokine receptors in predicting cancer metastasis and prognosis. J Exp Clin Cancer Res CR 29: 16 20181250 10.1186/1756-9966-29-16 PMC2845107

[bib27] Majka M, Janowska-Wieczorek A, Ratajczak J, Kowalska MA, Vilaire G, Pan ZK, Honczarenko M, Marquez LA, Poncz M, Ratajczak MZ (2000) Stromal-derived factor 1 and thrombopoietin regulate distinct aspects of human megakaryopoiesis. Blood 96: 4142–4151 11110685

[bib28] McShane LM, Altman DG, Sauerbrei W, Taube SE, Gion M, Clark GM, Statistics Subcommittee of the NCI-EORTC Working Group on Cancer Diagnostics (2005) REporting recommendations for tumour MARKer prognostic studies (REMARK). Nat Clin Prac Oncol 2: 416–422 10.1038/sj.bjc.6602678 PMC2361579 16106245

[bib29] Meiron M, Zohar Y, Anunu R, Wildbaum G, Karin N (2008) CXCL12 (SDF-1alpha) suppresses ongoing experimental autoimmune encephalomyelitis by selecting antigen-specific regulatory T cells. J Exp Med 205: 2643–2655 18852294 10.1084/jem.20080730 PMC2571938

[bib30] Miyanishi N, Suzuki Y, Simizu S, Kuwabara Y, Banno K, Umezawa K (2010) Involvement of autocrine CXCL12/CXCR4 system in the regulation of ovarian carcinoma cell invasion. Biochem Biophys Res Commun 403: 154–159 21059341 10.1016/j.bbrc.2010.11.007

[bib31] Muller A, Homey B, Soto H, Ge N, Catron D, Buchanan ME, McClanahan T, Murphy E, Yuan W, Wagner SN, Barrera JL, Mohar A, Verastegui E, Zlotnik A (2001) Involvement of chemokine receptors in breast cancer metastasis. Nature 410: 50–56 11242036 10.1038/35065016

[bib32] Nanki T, Lipsky PE (2000) Cutting edge: stromal cell-derived factor-1 is a costimulator for CD4 (+) T cell activation. J Immunol 164: 5010–5014 10799853 10.4049/jimmunol.164.10.5010

[bib33] Orimo A, Gupta PB, Sgroi DC, Arenzana-Seisdedos F, Delaunay T, Naeem R, Carey VJ, Richardson AL, Weinberg RA (2005) Stromal fibroblasts present in invasive human breast carcinomas promote tumor growth and angiogenesis through elevated SDF-1/CXCL12 secretion. Cell 121: 335–348 15882617 10.1016/j.cell.2005.02.034

[bib34] Parkin DM, Pisani P, Ferlay J (1999) Estimates of the worldwide incidence of 25 major cancers in 1990. Int J Cancer 80: 827–841 10074914 10.1002/(sici)1097-0215(19990315)80:6<827::aid-ijc6>3.0.co;2-p

[bib35] Peled A, Petit I, Kollet O, Magid M, Ponomaryov T, Byk T, Nagler A, Ben-Hur H, Many A, Shultz L, Lider O, Alon R, Zipori D, Lapidot T (1999) Dependence of human stem cell engraftment and repopulation of NOD/SCID mice on CXCR4. Science 283: 845–848 9933168 10.1126/science.283.5403.845

[bib36] Poznansky MC, Olszak IT, Foxall R, Evans RH, Luster AD, Scadden DT (2000) Active movement of T cells away from a chemokine. Nat Med 6: 543–548 10802710 10.1038/75022

[bib37] Salcedo R, Oppenheim JJ (2003) Role of chemokines in angiogenesis: CXCL12/SDF-1 and CXCR4 interaction, a key regulator of endothelial cell responses. Microcirculation 10: 359–370 12851652 10.1038/sj.mn.7800200

[bib38] Samara G, Lawrence D, Chiarelli C, Chlarelli C, Valentino M, Lyubsky S, Zucker S, Vaday G (2004) CXCR-4-mediated adhesion and MMP-9 secretion in head and neck squamous cell carcinoma. Cancer Lett 214: 231–241 15363550 10.1016/j.canlet.2004.04.035

[bib39] Schimanski CC, Galle PR, Moehler M (2008) Chemokine receptor CXCR4-prognostic factor for gastrointestinal tumors. World J Gastroenterol 14: 4721–4724 18720530 10.3748/wjg.14.4721 PMC2739331

[bib40] Scotton CJ, Wilson JL, Milliken D, Stamp G, Balkwill FR (2001) Epithelial cancer cell migration: a role for chemokine receptors? Cancer Res 61: 4961–4965 11431324

[bib41] Scotton CJ, Wilson JL, Scott K, Stamp G, Wilbanks GD, Fricker S, Bridger G, Balkwill FR (2002) Multiple actions of the chemokine CXCL12 on epithelial tumor cells in human ovarian cancer. Cancer Res 62: 5930–5938 12384559

[bib42] Soslow RA (2008) Histologic subtypes of ovarian carcinoma: an overview. Int J Gynecol Pathol 27: 161–174 18317227 10.1097/PGP.0b013e31815ea812

[bib43] Sun Y, Fang M, Wang J, Cooper C, Pienta K, Taichman R (2007) Expression and activation of alpha v beta 3 integrins by SDF-1/CXC12 increases the aggressiveness of prostrate cancer cells. Prostate 67: 61–73 17034033 10.1002/pros.20500

[bib44] Sun Y, Wang J, Shelburne C, Lopatin D, Chinnaiyan A, Rubin M, Pienta K, Taichman R (2003) Expression of CXCR4 and CXCL12 (SDF-1) in human prostrate cancers (PCa) *in vivo*. J Biochem 89: 462–473 10.1002/jcb.10522 12761880

[bib45] Taichman R, Cooper C, Keller E, Pienta K, Taichman N, McCauley L (2002) Use of the stromal cell-derived factor-1/CXCR4 pathway in prostrate cancer metastasis to bone. Cancer Res 62(6): 1832–1837 11912162

[bib46] Vianello F, Papeta N, Chen T, Kraft P, White N, Hart W, Swart A, Rhee S, Palu G, Irmia D, Toner M, Weissleder R, Poznansky M (2006) Murine B16 melanomas expressing high levels of the chemokine stromal-derived factor-1/CXCL12 induce tumour-specific T cell chemorepulsion and escape immune control. J Immunol 176: 2902–2914 16493048 10.4049/jimmunol.176.5.2902

[bib47] Wang L, Yang B, Yang Q, Qiao S, Wang Y, Sun Y (2009) Strong expression of chemokine receptor CXCR4 by renal cell carcinoma cells correlates with metastasis. Clin Exp Metastasis 26: 1049–1054 19859817 10.1007/s10585-009-9294-3

[bib48] Wang N, Wu Q, Fang Y, Mai H, Zeng M, Shen G, Hou J, Zeng Y (2005) Expression of chemokine receptor CXCR4 in nasopharyngeal carcinoma: pattern of expression and correlation with clinical outcome. J Transl Med 3: 26 15978137 10.1186/1479-5876-3-26 PMC1188078

[bib49] Zhou N, Luo Z, Luo J, Liut D, Hall J, Pomerantz R, Huang Z (2001) Structural and functional characterization of human CXCR4 as a chemokine receptor and HIV-1 co-receptor by mutagenesis and molecular modeling studies. J Biol Chem 276: 42826–42833 11551942 10.1074/jbc.M106582200

